# Retraction Note: The effect of soil nutrients and moisture during ontogeny on apparent wood density of *Eucalyptus grandis*

**DOI:** 10.1038/s41598-021-91048-y

**Published:** 2021-05-27

**Authors:** Vinicius Resende Castro, Roger Chambi-Legoas, Mario Tommasiello Filho, Paula Gabriella Surdi, José Cola Zanuncio, Antonio José Vinha Zanuncio

**Affiliations:** 1 Universidade Federal de Viçosa, Departamento de Engenharia Florestal, Viçosa, 36570-900 Brasil; 2 Escola Superior de Agricultura, Luiz de Queiroz, Departamento de Engenharia Florestal, Piracicaba, 13418-900 Brasil; 3 Universidade Federal de Viçosa, Departamento de Entomologia/BIOAGRO, Viçosa, 36570-900 Brasil; 4 Universidade Federal de Uberlândia, Instituto de Ciências Agrárias-ICIAG, Monte Carmelo, 38500-000 Brasil

Retraction of *Scientific Reports* 10.1038/s41598-020-59559-2, published online 13 February 2020

The Editors have retracted this article.

After publication it was brought to the Editors’ attention that this article reports data that have previously been published^1^, specifically the data for 12, 24 and 36 months shown in Table 1, Table 2 and Figure 2. The Editors therefore consider this article to be redundant.

All authors agree to this retraction.
